# Work engagement influencing factors of Japanese midwives working in prenatal medical centers

**DOI:** 10.18332/ejm/183805

**Published:** 2024-03-11

**Authors:** Yuki Fukuya, Yoko Asaka

**Affiliations:** 1 Department of Maternal and Child Nursing/Midwifery, Course of Nursing, Graduate School of Medicine, Mie University, Tsu, Japan

**Keywords:** midwife, Japan, professional identity, work engagement, perinatal medical center

## Abstract

**INTRODUCTION:**

There have been significant changes in the work environment of midwives, such as the establishment of prenatal medical centers and shared wards for obstetrics and other departments. These changes in the work environment pose a psychological burden on midwives. Under these circumstances, the concept of work engagement is essential for overcoming the ensuing difficulties. However, no studies have yet investigated midwives working in prenatal medical centers in Japan. This study examined the factors affecting work engagement among midwives working in prenatal medical centers in Japan.

**METHODS:**

A cross-sectional survey of 498 midwives working at prenatal medical centers nationwide was conducted. Data were analyzed using the Mann-Whitney U test or Kruskal-Wallis test, correlation analysis using Spearman’s rank correlation coefficient, and multiple regression analysis as linear regression.

**RESULTS:**

The median of work engagement score was 3.00 (IQR: 2.40–3.60). The multiple linear regression analysis showed that professional identity (β=0.494, p<0.001), resilience (β=0.243, p<0.001), presence of in-hospital midwifery in the facility (β=0.138, p<0.011), and age (β=0.085, p=0.021) were significant. The adjusted R^2^ value was 0.490 (p<0.001).

**CONCLUSIONS:**

These results offer management insights into improving work engagement among midwives working in prenatal medical centers in Japan. The proposed measures include hospital/ward administrators establishing the professional identity of midwives and providing them with opportunities to demonstrate their expertise, for instance, within in-hospital midwifery systems at perinatal medical centers.

## INTRODUCTION

In Japan, ‘midwife’ refers to a woman licensed by the Minister of Health, Labor, and Welfare, who practices midwifery and provides healthcare to pregnant women, women in the postpartum period, and newborn. The primary role of midwives is to provide care for normal childbirth using professional diagnoses and techniques. However, their work is not limited to childbirth and related perinatal care, but includes also sexual, reproductive, maternal, newborn, and adolescent health (SRMNAH) and sex education. Midwives can work not only in hospitals and clinics but also open and manage midwifery centers in local communities^1^.

With the decline in birth rates, the number of delivery facilities in Japan is decreasing. However, progress has been made in the development of the perinatal medical system, with perinatal medical centers established in each prefecture of Japan^1^. As a result, 32% of all delivery facilities are perinatal medical centers, whose primary role is to provide advanced and emergency medical care for high-risk cases^1^. Moreover, approximately 80% of the delivery facilities, including perinatal medical centers, are shared obstetric inpatient wards with patients from other departments, which is known as a shared ward in Japan^2^.

This work environment poses a psychological burden on midwives working in prenatal medical centers. In Japan, a midwife license is required to acquire certification as a registered nurse^1^. Thus, it was reported that midwives working in shared wards in hospitals experienced dilemmas and stress when taking on the duties of a nurse and were unable to fully demonstrate their expertise as a midwife^3-5^. In addition, midwives working in perinatal medical centers experience anxiety, fear, ethical conflicts, and trauma because of their frequent contact with critically ill obstetric patients^6^. This makes it difficult to maintain one’s identity as a midwife^5^ and is likely to affect job satisfaction^7^.

The number of midwives in Japan has increased over the past decade, from 32480 in 2010 to 40632 in 2019, with those working in hospitals accounting for approximately 60% of the total number^8^. There were approximately 11000 midwives in the perinatal medical centers^9^, which accounts for 44.5% of all hospital midwives. Thus, it is presumed that a considerable number of midwives have difficulty in performing midwifery tasks.

Under these circumstances, the concept of work engagement is important for addressing the ensuing difficulties. Work engagement was proposed by Schaufeli et al.^10^ and is defined as ‘a positive, fulfilling, work-related state of mind, which is characterized by vigor, dedication, and absorption’. Work engagement is positively correlated with nurses’ job satisfaction and patient satisfaction, and negatively correlated with nurses’ turnover^11-13^.

The Job Demands-Resource Model (JD-R model) is commonly used as a key concept of work engagement^11^. The JD-R model explains the motivational process through which job and personal resources enhance work engagement and create positive attitude and behaviors toward work and the organizations^10^. Work engagement increases when job resources, such as their work environment^14^, presence of role models, and physician support^15^ are appropriate, regardless of the level of work demand. Regarding personal resources, studies have shown positive relationships between nurses’ and midwives’ work engagement and self-efficacy^15^, proactive personality^13^, self-motivation^16^, work values^17^, and resilience^18-20^.

The Nursing Job Demands-Resources (NJD-R) model, proposed by Keyko^21^, is more suitable for explaining work engagement in nursing practices. In this model, professional practice and its environment, autonomy, role, and identity were proposed as professional resources. However, studies have not yet investigated midwives working in prenatal medical centers in Japan.

We propose the following hypotheses based on the N-JDR model: 1) professional identity, as a professional resource, has a positive association with work engagement; 2) resilience, as a personal resource, has a positive association with work engagement; and 3) work environment, as a job resource, which includes ward composition, midwifery outpatient clinic, in-hospital midwifery system, is also positively associated with work engagement.

Professional identity is described as the self-concept and perception of one’s profession and working ability^22^ and is developed through an individual’s growth, maturity, and socialization^23^. Previous studies have shown the interconnection between professional identity and work engagement among registered nurses^24-26^. Therefore, professional identity is assumed to be positively related to work engagement.

Resilience refers to an individual’s capacity to adapt and recover from adversity, trauma, or significant stress and the ability to maintain stable functioning, meet challenges, and prevail in the face of adversity^27^. High levels of resilience have been cited as essential for nurses to cope with workplace stress. Previous nursing studies reported that resilience partially mediated the relationship between work-related stress and burnout of nurses and nursing students^18^; it was also negatively associated with stress, burnout, posttraumatic stress disorder, workplace bullying^19^, as lower levels of resilience lead to higher turnover intention in hemodialysis nurses^20^. However, to our knowledge, the relationship between work engagement and resilience among midwives has not been investigated.

In this study, the work environment was viewed as an organizational structure that influenced midwives’ ability to exercise their professional expertise. Working in a shared ward reduced midwives’ job satisfaction^4^. In contrast, work engagement is positively correlated with job satisfaction^11^. Thus, a shared ward, a type of ward composition, could be a work environment negatively related to midwives’ work engagement. Hospitals and clinics have introduced midwifery outpatient clinics and in-hospital midwifery system in Japan^1^. Midwifery outpatient clinics are systems wherein midwives, in cooperation with physicians, assess the normal progress of pregnancy and provide health check-ups and guidance independently^1^. In-hospital midwifery systems include outpatient midwifery functions and midwife-led care during labor. Midwives working in these systems are reported to have high levels of satisfaction with their work because they can provide continuous care to the patients^28^. Thus, these two systems were assumed to be positively related to work engagement.

This study examined the influence of professional identity, resilience, and work environment on work engagement among midwives working in perinatal medical centers in Japan.

## METHODS

This study used a cross-sectional design. The study participants were midwives who worked at prenatal medical centers nationwide and were enrolled in the obstetrics department. Ward managers were excluded from the study. We conducted a questionnaire survey of midwives working in 408 prenatal medical centers. The calculated sample size was 385, with an error of 5%, a reliability of 95%, and a population rate of 50%. To secure 385 participants, we distributed 800 questionnaires based on a 50% response rate in previous studies^15^.

First, we reached out to nursing directors in 408 hospitals with perinatal medical centers asking for their participation and the number of questionnaires to be sent when cooperating with the research. Nursing directors from 165 of the 408 hospitals agreed to participate. We sent self-administered and anonymous questionnaires, explanatory documents, and individual self-addressed envelopes to the midwives in the participating facilities and asked each director to distribute them to eligible midwives. Completed questionnaires were mailed to the researchers using individual self-addressed envelopes. The questionnaire was distributed to 1255 midwives, and 498 questionnaires were returned, accounting for a response rate of 43.2%). The survey was conducted from April to September 2022.

### Measures

The questionnaire consisted of scales that had already been developed for work engagement, professional identity, and resilience, and questions about the work environment were selected by the authors.

### Work engagement

Work engagement was assessed using the 9-item Japanese version of the Utrecht Work Engagement Scale (UWES-J), originally developed by Schaufeli et al. ^29^. This scale has been commonly used in previous nursing studies and consists of 3 subscales: vigor, dedication, and absorption, which are rated on a 7-point Likert scale ranging from 7 (strongly agree) to 1 (strongly disagree). Higher UWES-J scores indicate higher work engagement. The Cronbach’s α coefficient for the 9 items was 0.92^30^ and 0.91 in this study.

### Professional identity

Professional identity was assessed using A Midwife Occupational Identity Scale developed by Sato et al.^31^. Occupational identity of midwives was defined as ‘the self-identity of being a midwife and the recognition of the meaning and value of working professionally as a midwife’^31^. Thus, we considered this scale as a suitable measure of the professional identity of the study participants. It consists of 23 items with five factors. It consists of items, such as: ‘confidence in what is needed as a midwife’, ‘establishing one’s own view of midwifery’, ‘confidence in choosing a midwife’, ‘confidence in the professionalism of midwives’, and ‘intention to contribute to society as a person’. Responses were obtained on a 7-point Likert scale ranging from 7 (strongly agree) to 1 (strongly disagree); the higher the score, the higher the professional identity. The reliability of this scale was 0.79, indicating sufficient internal consistency, and its construct validity was confirmed through factor analysis^32^. The Cronbach’s α coefficient for the entire scale in this study was 0.81.

### Resilience

Resilience was measured using the Nurse Resilience Scale, developed by Ihara^33^, which consists of four subscales: a ‘positive approach to nursing’, ‘interpersonal skills’, ‘ability to deal with novelty’, and ‘private support’. Responses were obtained on a 5-point Likert scale ranging from 5 (yes) to 1 (no), with possible total scores ranging from 32 to 160. Higher scores reflected higher resilience. Cronbach’s alpha coefficient was 0.84, and those of the subscales were between 0.63 and 0.87^33^. Although Cronbach’s alpha coefficient for the ‘ability to deal with novelty’ was 0.63, indicating low internal consistency, it is considered to be within an acceptable range. The Cronbach’s α coefficient for the entire scale in this study was 0.85.

### Work environment

In this study, ward composition and whether the facilities had midwifery outpatient clinics and in-hospital midwifery systems, were selected as variables for the work environment. Regarding ward composition, the participants were asked to choose one from obstetrics, obstetrics and gynecology, and obstetrics and other departments. The participants were also asked whether they had care experiences providing in midwifery outpatient clinics or in-hospital midwifery system.

### Participant characteristics

The participants’ demographic characteristics included age, marital status, whether they had children, work years as a midwives, the number of attendances in a vaginal birth, and whether they were Clinical Ladder of Competencies for Midwifery Practice (CLoCMiP) certified, which is the certification that midwives with more than five years of clinical experience are eligible to acquire^1^.

### Statistical analysis

First, a descriptive analysis of the participants’ characteristics and outcome variables (UWES-J) was conducted using the Mann-Whitney U test or Kruskal–Wallis test, and Bonferroni’s method was used for multiple comparisons. Second, bivariate analyses were conducted using Spearman’s rank correlation coefficient between the UWES-J and the scale scores of professional identity, resilience, and participant characteristics (age, work years as a midwife, and number of attendances in a vaginal births). Third, multiple linear regression analysis using the forced-entry method was conducted. The UWES-J was used as the dependent variable, and age, professional identity, resilience, in-hospital midwifery in the facility (absence=0, presence=1), outpatient midwifery in the facility (absence=0, presence=1), and ward composition (obstetrics shared with gynecology and other departments=0, obstetrics=1) as independent variables. A nominal scale is used as the dummy variable. The variance inflation factor was used to diagnose multicollinearity. In addition, because the variance inflation factor for age and work years as a midwife was ≥4, we entered age considering multicollinearity. The statistical software IBM SPSS, version 27.0, (SPSS Inc., Chicago, IL, USA) was used for data analysis, and a p<0.05 was considered statistically significant.

### Ethical considerations

The researchers clarified cooperation in the study with documents outlining the purpose and methods of the study, the voluntary nature of participation, and the right to refuse participation. Written consent for study cooperation was obtained from the participants who were attached to the first page of the questionnaire. This study was approved by the Research Ethics Committee of Mie University (No. U2022-010).

## RESULTS

The average age of the participants was 36.2 ± 9.2 years, and the largest group of 169 (35.1%) were aged 30–39 years. Nearly half of the participants, 238 (49.5%) had a spouse, and 196 (40.8%) had children.

The average of work years as a midwife was 11.0 ± 7.9 years, with 145 (30.1%) having >15 years of experience, followed by 130 (27.0%) having <10 years. There were 182 participants (37.9%) working in an obstetrics inpatient ward and 168 (35.0%) in a shared ward with obstetrics and gynecology. Furthermore, 130 (27.1%) worked in obstetrics and other departments, including internal medicine, pediatrics, urology, otolaryngology, and emergency department. Of the 443 respondents who answered the number of births assisted, the average number was 220, with 162 (36.6%) answering 0–99, followed by 111 (25.3%) who answered 100–199.

The median of the UWES-J score was 3.00 (IQR: 2.40–3.60). The Mann-Whitney U test showed that the UWES-J scores of midwives who were married (p<0.001), had children (p=0.031), had acquired CLoCMiP level III (p<0.001), and were present in the in-hospital midwifery system (p=0.011), were significantly higher than those of midwives who were not. No significant differences were found in the midwifery outpatient clinics (Table 1).

**Table 1:** UWES-J score of the participants by demographic and work environment variables

*Variables*	*Categories*	*n*	*Median*	*IQR*	*p*	
**Marital status^b^**	Married	246	3.10	2.30–3.40	<0.001***	
	Unmarried	252	2.90	2.60–3.70		
**Have children^b^**	Yes	203	3.00	2.60–3.70	0.031*	
	No	294	2.90	2.30–3.60		
**CLoCMiP level III^b^**	Acquired	159	3.30	2.70–3.90	<0.001***	
	Non-acquired	339	2.90	2.30–3.40		
**Ward composition^a^**	1. Obstetrics	191	3.10	2.60–3.70	0.022*	
	2. Obstetrics and gynaecology	173	2.90	2.30–3.35		1-2 0.006**
	3. Obstetrics and other	133	2.90	2.40–3.70		1-3 0.108
**Midwifery outpatient clinic^b^**	Presence	377	3.00	2.40–3.60	0.962	
	Absence	117	3.00	2.50–3.70		
**In-hospital midwifery system^b^**	Presence	113	3.10	2.70–3.80	0.011*	
	Absence	382	2.90	2.38–3.60		

a Kruskal-Wallis test was performed, and Bonferroni’s method was used for multiple comparisons.

b Mann-Whitney U test was performed.

CLoCMiP is a clinical ladder of competencies for midwifery practice. IQR: interquartile range.

* p<0.05,

** p<0.01,

*** p<0.001.

The Kruskal-Wallis test showed significant differences between the groups of the UWES-J of ward composition (p=0.022). Multiple comparisons revealed a significant difference in ward composition between obstetrics and obstetrics and gynecology (p<0.006), and UWES-J showed that midwives working in obstetrics had a significantly higher score than midwives working in obstetrics and gynecology (Table 1).

Spearman’s rank correlation coefficients between the UWES-J score, professional identity, resilience, and demographic variables, were calculated. The UWES-J score was significantly correlated with professional identity (r=0.656, p<0.001), resilience (r=0.573, p<0.001), age (r=0.176, p<0.001), work years as a midwife (r=0.204, p<0.001), and the number of attendances in a vaginal birth (r=0.179, p<0.001).

Table 2 shows the results of the multiple linear regression analysis of the influencing variables associated with UWES-J scores. Professional identity (β=0.494, B=0.025, 95% CI: 0.021–0.029, p<0.001), resilience (β=0.243, B=0.031, 95% CI: 0.021–0.042, p<0.001), presence of in-hospital midwifery in the facility (β=0.138, B=0.315, 95% CI: 0.159–0.471, p<0.011), and age (β=0.085, B=0.009, 95% CI: 0.002–0.016, p=0.021) were significant. The adjusted R^2^ value, which is the overall explanatory rate of the regression equation, was 0.490 (p<0.001). The standardized coefficient (β) results showed that professional identity had the greatest influence on the UWES-J scores.

**Table 2:** Multiple linear regression analysis of influencing factors associated with UWES-J score (N=498)

*Factors*	*B (95% CI)*	*SE*	*β*	*t*	*p*	*VIF*
Total professional identity score	0.025 (0.021–0.029)	0.002	0.494	11.593	<0.001***	1.705
Total resilience score	0.031 (0.021–0.042)	0.005	0.243	5.732	<0.001***	1.681
Ward composition	0.035 (-0.030–0.099)	0.033	0.035	1.057	0.291	1.034
In-hospital midwifery	0.315 (0.159–0.471)	0.079	0.138	3.965	<0.001***	1.135
Midwifery outpatient clinic	-0.085 (-0.236–0.067)	0.077	-0.038	-1.099	0.272	1.131
Age (years)	0.009 (0.002–0.016)	0.003	0.085	2.554	0.021**	1.039

F=77.288, p<0.001, R^2^=0.497; adjusted R^2^=0.490. The dependent variable was the UWES score. B: unstandardized coefficient. β: standardized coefficient. SE: standard error.

* p<0.05,

** p<0.01,

*** p<0.001.

## DISCUSSION

The UWES-J score obtained in this study was lower than that reported in previous studies on Japanese midwives^11,15^. Ward managers were excluded from this study, because the higher positions were related to a higher degree of work engagement^34^. Thus, the UWES-J score in this study was slightly lower than those reported in previous studies.

The multiple linear regression analysis in this study showed that approximately 50% of the UWES-J score of midwives working at perinatal medical centers could be explained by professional identity, resilience, the in-hospital midwifery system, and age.

The results showed that professional identity had the greatest influence on the UWES-J scores among midwives working at prenatal medical centers, which supports the findings of previous studies^24-26^ showing an interconnection between professional identity and work engagement. Nurses with a higher professional identity have a stronger sense of professional benefits and experience more pride, efficacy, and sense of accomplishment in the workplace^26^. Nurses with a high professional identity provide higher quality care to patients and improve their care skills, which leads to higher satisfaction in their nursing profession and a lower chance of turnover^35^. As for midwives, their inability to demonstrate their expertise makes it difficult to maintain their identity as midwives and reduces work motivation^7^. Thus, midwives with high levels of identity were more enthusiastic about their work. Professional identity is a variable included in the professional resources in the N-JDR model^21^. The results confirm that professional identity has a positive relationship with work engagement among Japanese midwives.

In addition, previous research has reported that a high level of career identity leads to career success and that nurses’ work engagement is a mediating variable^25^. In other words, enhancing professional identity increases work engagement, leading to career success. Further research is necessary to examine the relationships among professional identity, work engagement, and career success among midwives.

The results of this study are consistent with those of previous studies which highlight that resilience is associated with work engagement^18-20^. High levels of resilience have been cited as essential for nurses to cope with workplace stressors, and those with high resilience tend to have positive emotions, even in difficult situations^27^. Therefore, resilience is an important factor for alleviating occupational stress, preventing burnout, and enabling midwives to be actively involved in their work. Previous research has considered personal resources as a factor increasing work engagement^32^. This study also confirmed that resilience, an individual resource, is one of those factors.

In addition, the results of this study revealed that the UWES-J score of midwives working in-hospital midwifery system was significantly higher, which suggests that working in a setting where midwife-led care, not limited to prenatal check-ups and health guidance but also attendance at a vaginal birth, leads to higher work engagement. Thus, midwives working in perinatal medical centers, even in shared wards, can enhance their work engagement if the workplace allows them to fully demonstrate their expertise.

Although multiple regression analysis showed no significant relationship between ward composition and UWES-J scores, there were significant differences in UWES-J scores depending on ward composition. The scores of midwives working in obstetrics were significantly higher than those of midwives working in obstetrics and gynecology. The results of this study are consistent with previous studies^4^, which reported that the difficulty midwives face in demonstrating their expertise in shared wards is related to burnout. Therefore, being responsible for gynecological patients and midwifery care simultaneously, could be a factor that inhibits midwives’ work engagement.

Age showed a significant positive correlation with the UWES-J score in the multiple regression analysis, which is consistent with previous studies^17,36^. Also, a study related to burnout, which is a concept opposite to work engagement^10^, reported that nurses aged >40 years with >11 years of experience tended to be less prone to burnout^4^. A previous study of nurses also reported that those aged ≥40 years had higher UWES-J scores than those in younger age groups^36^. Furthermore, lack of experience was related to lower work engagement^4^. Thus, improvements in skills and attitudes with age and years of experience positively affect work engagement.

The results of this study proved the hypothesis proposed based on the N-JDR model. To increase the work engagement of midwives in perinatal medical centers in Japan, it is necessary to create a work environment that fosters and supports their professional identities.

### Limitations

This study has several limitations. First, due to its cross-sectional nature, the observed associations between variables may not be causal. Second, this study did not compare the midwives’ work engagement across facilities. Third, this study did not examine the performance or quality of care as outcomes. Fourth, this study did not investigate midwives’ work patterns, which may influence work engagement and job satisfaction. Fifth, the response rate in this study (43.2 %) was slightly lower than the 50% in a similar previous study^15^. This is because some midwives may have been missed, as the questionnaire was distributed to the study participants by the director of each facility. Nevertheless, the results of this study are considered significant because they were examined based on a larger number of responses than those calculated by sample size calculations. Finally, to improve work engagement, it is necessary to consider specific approaches that individuals and organizations should adopt in the future.

## CONCLUSIONS

This study examined the factors affecting work engagement among midwives working in prenatal medical centers in Japan. We observed the highest level of professional identity in work engagement, followed by resilience, in-hospital midwifery, and age. These results provide suggestions for management to improve work engagement among midwives in prenatal medical centers in Japan.

## Data Availability

The data supporting the findings of this study are available from the corresponding author upon request.
